# Depression and Cardiovascular Disease: The Viewpoint of Platelets

**DOI:** 10.3390/ijms21207560

**Published:** 2020-10-13

**Authors:** Patrizia Amadio, Marta Zarà, Leonardo Sandrini, Alessandro Ieraci, Silvia Stella Barbieri

**Affiliations:** 1 Unit of Brain-Heart Axis: Cellular and Molecular Mechanism, Centro Cardiologico Monzino IRCCS, 20138 Milan, Italy; marta.zara@ccfm.it (M.Z.); leonardo.sandrini@ccfm.it (L.S.); 2 Laboratory of Neuropsychopharmacology and Functional Neurogenomics, Department of Pharmaceutical Sciences, University of Milan, 20133 Milan, Italy; alessandro.ieraci@unimi.it

**Keywords:** platelets, depression, catecholamines, adipokines, low density lipoproteins, reactive oxygen species, chemokines

## Abstract

Depression is a major cause of morbidity and low quality of life among patients with cardiovascular disease (CVD), and it is now considered as an independent risk factor for major adverse cardiovascular events. Increasing evidence indicates not only that depression worsens the prognosis of cardiac events, but also that a cross-vulnerability between the two conditions occurs. Among the several mechanisms proposed to explain this interplay, platelet activation is the more attractive, seeing platelets as potential mirror of the brain function. In this review, we dissected the mechanisms linking depression and CVD highlighting the critical role of platelet behavior during depression as trigger of cardiovascular complication. In particular, we will discuss the relationship between depression and molecules involved in the CVD (e.g., catecholamines, adipokines, lipids, reactive oxygen species, and chemokines), emphasizing their impact on platelet activation and related mechanisms.

## 1. Introduction

Cardiovascular disease (CVD), still the most common cause of death worldwide [WHO, The Top 10 Causes of Death, https://www.who.int/news-room/fact-sheets/detail/the-top-10-causes-of-death, Accessed date: 20 June 2019], remains the major target for public health efforts. The association between psychosocial factors and CVDs has long been recognized, and a recent meta-analysis of prospective epidemiological studies found that psychological factors predict cardiovascular morbidity and mortality [1,2]. Specifically, depression has been associated with coronary heart disease (CHD) [3,4], and with atrial fibrillation [5,6]. After acute coronary syndrome (ACS), depression is a risk factor for all cause and cardiac mortality, as well as for composite outcomes including mortality or non-fatal cardiac events [7]. However, the relationship between depression and CVD is multifaceted and bidirectional: not only depression may increase the risk of CVD, but also cardiovascular events may increase the risk of depression [8]. The vast majority of studies are not able to determine whether this association is causative or temporally related, raising the eternal chicken-and-egg dilemma.

Behavioral factors, autonomic dysregulation, activation of the hypothalamic–pituitary–adrenal (HPA)-axis, inflammatory response [3], oxidative stress [9], serotoninergic and neurotrophins pathway dysregulation [10,11,12], endothelial dysfunction and platelet activation [3] are the proposed mechanisms underlying this relationship.

Among these mechanisms, the platelet activation is one of the most attractive, seeing platelets as a potential mirror of the brain (dys)-function [13]. Indeed, even though platelet and neurons are basically different cells, they share common characteristics in subcellular organization [14] and in protein composition [15,16,17,18,19,20,21], representing as consequence an alternative tool to investigate neuronal dysfunction as well as a peripheral tracer of the onset and progression of brain-related pathologies.

Moreover, the platelet hyper-reactivity could at least partially explain the increased vulnerability of depressed patients to acute thrombotic event and ischemic heart disease [22], as well as their increased mortality post-myocardial infarction [23].

**Figure 1:**
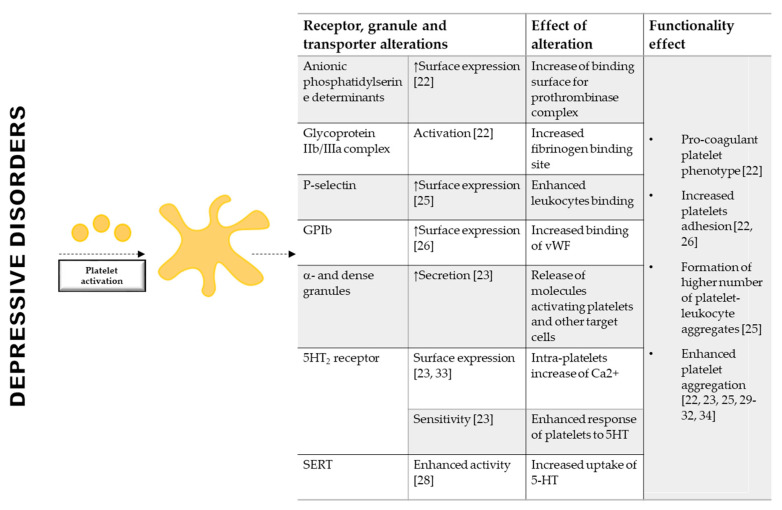
Platelets of depressed patients show hyper-reactivity. Patients with depression, show hyper-reactive platelets. Indeed, platelets of depressed patients display a greater exposure of anionic phosphatidylserine determinants, an increased activation of Glycoprotein IIb/IIIa, a higher expression of P-selectin and Glycoprotein Ib, an increased expression and sensitivity of 5HT_2_ receptor, an enhanced activity of SERT and an enhanced aggregation and sensitivity in response to collagen thrombin, and serotonin. Platelet hyper-reactivity is also confirmed by a greater platelet granules secretion. GP: Glycoprotein; vWF: von Willebrand Factor; 5-HT: 5-Hydroxytriptamine; 5-HT_2_: 5-HT receptor; SERT: plasma membrane serotonin transporter. ↑: increased compared to control.

Of note, platelets of depressed patients display a greater exposure of anionic phosphatidylserine determinants, an increased activation of glycoprotein (GP) IIb/IIIa [22], a greater granules secretion [24], a higher expression of P-selectin and GPIb [25,26], and an enhanced aggregation in response to collagen and thrombin compared to control subjects [22,27], whereas platelet aggregation is unchanged when Adenosine Diphosphate (ADP) and TRAP1-6 were used [22,26,27] (Figure 1). An extensively investigated molecule in the relation between depression and platelets activation is the serotonin (5-HT) [28,29]. Platelets share with serotoninergic neurons several similarities in 5-HT uptake, storage, metabolism and release mechanisms, representing a good surrogate to study neuropsychiatric research [30]. Of note, platelets from depressed patients show a greater aggregation in response to 5-HT [31,32,33], display enhanced platelet serotonin uptake [30], that favors platelet response to ADP [34], and an increased platelet 5-HT_2_ receptor binding and density [35,36] (Figure 1). Despite the already proved importance of 5-HT in this contest also other circulating molecules may be involved.

This review will be focused on the prothrombotic state of patients affected by depressive disorders. In particular, it will analyze the impact of catecholamines, adipokines, lipids, reactive oxygen species and chemokines, in the pathophysiological link between depression and CVD, emphasizing the critical role of platelet activation and the related molecular mechanisms.

## 2. Catecholamines

The catecholamines are adaptive and maladaptive stress hormones; they activate behavioral and physiological processes facilitating the overcoming of stress [37]. Endogenous catecholamines include dopamine (DA), noradrenaline (norepinephrine/NE), and adrenaline (epinephrine/EPI) [37]. Catecholamines, produced and released by the sympathetic system, brain and adrenal medulla [37], exert their effects on multiple organs/compartments [38]. Although catecholamines are essential constituents of physiologic cardiovascular regulation, their effects are greatly emphasized by abnormal conditions [39].

The response to EPI and NE is mediated by a set of G protein-coupled adrenergic receptors (ARs), α and/or β-adrenergic receptors, that are targets for several cardiovascular drugs [37,40]. DA receptors are all members of the G protein-coupled receptor family and they are divided into two subtypes: D1-like receptors coupled with Gs alpha subunit (Gs) (D1 and D5) and D2-like receptors coupled with Gi alpha subunit (Gi) (D2, D3, D4). NE, EPI and DA have a prominent position in the pathogenic mechanisms of several cardiovascular disorders, such as angina pectoris, heart failure, arterial hypertension, atherosclerosis and thrombosis [39,41].

### 2.1. Catecholamines in Depression

The discovery, in the 1960s, that the inhibition of neuronal uptake of NE, the primary target for tricyclic antidepressants, reduced depressive symptoms, led to hypothesize and then to show that a deficit in catecholamine transmission could account for the depression [42]. On the other hand, the contribution of DA was largely neglected until few years ago.

Beyond alterations in adrenergic and dopaminergic receptors availability, and the consequent modification in the downstream pathways in the brain [43,44,45,46], depressive disorders have been also associated with changing in peripheral levels of catecholamines.

In spite of the scarce and outdated studies, plasma levels of EPI and NE result increased in depressed patients and their levels correlate with the severity of the pathology [47,48]. In agreement with previous data, more recent studies showed that patients suffering from depression and other major affective disorders have increased urinary levels of EPI, NE and DA [49], and the existence of a positive association between urinary EPI or NE and depressive symptoms [50] (Table 1).

All these findings provide the evidence that alterations in peripheral catecholamines levels may be relevant also in depression and not only in stress response, and pave the way to the potential link among catecholamines, depression and CVDs.

### 2.2. Catecholamines and Platelet Function

Since human platelets express both adrenergic and dopaminergic receptors [51,52,53], the high catecholamine levels may easier explain the association between depression and CVD. Basically, through platelet α2-adrenergic or dopaminergic receptors, they modulate thrombopoiesis [54,55], and platelet function [56,57,58]. Low concentrations of catecholamines and dopamine potentiate the effects of other agonists (e.g., ADP, collagen, and thrombin) enhancing platelet aggregation, whereas at high concentrations are sufficient alone to induce human platelet aggregation, granule secretion, and release of platelet markers (e.g., Platelet Factor 4 (PF4) and β-thromboglobulin (BTG) [59,60,61] (Table 1).

Specifically, the effects of EPI on human platelet activation has been extensively investigated in vitro providing the evidence that in platelet α2-adrenergic receptors are selectively coupled to Gz family members but not to Gq or G12 family members [62]. The activation of Gz mediated by EPI, inhibits cyclic Adenosine Monophosphate (cAMP) formation and promotes the activation of Rap1B and PI 3-kinase [63], enhancing the effects of other agonists. Interestingly, EPI, not affecting Phospholipase C (PLC), is unable to cause platelet shape change [64].

Remarkable, EPI infusion induces a threefold increase of platelet thromboxane (TX) production [65], and enhances platelet fibrinogen binding and platelet aggregation induced by thrombin [66].

In vivo infusion or in vitro exposure to EPI, enhances ADP-induced platelet aggregation and clot formation both in healthy subjects treated with ticagrelor and in ACS patients under acetylsalicylic acid and ticagrelor therapy [67,68] (Table 1).

**Table 1:** Catecholamines levels in MDD and effect on megakaryocytes and platelets.

Catecholamines in Depression and Platelets				
DEPRESSION		EFFECT ON MEGAKARYOCYTES		
**Stimulus**	**Levels**	**Stimulus**	**Receptor**	**Effect**
EPI NE	Increased circulating and urinary levels [47,48,49]	EPI NE	α-2-adrenoceptor	Megakaryocyte adhesion and migration [55] Pro-platelets formation [55]
DA	Increased urinary levels [49]	DA	D1/D2	Megakaryocytes differentiation [54]
		EFFECT ON PLATELETS		
**Stimulus**	**Levels**	**Stimulus**	**Receptor**	**Effect**
EPI NE	Increased circulating and urinary levels [47,48,49]	EPI NE	α-2-adrenoceptor	Low concentrations: Increase the sensitivity to collagen, thrombin and ADP [52,56,57,60]
		EPI	α-2-adrenoceptor	High concentrations: Induce aggregation alone [57,66] Increase TX production [65] Enhance fibrinogen binding [66] Induce clot formation [65,66,67]
DA	Increased urinary levels [49]	DA	D2 (?)	Low concentrations: Increase sensitivity to ADP [61]
			D2-like receptor	Induce platelet microaggregation [58] Induce platelet adhesion [58]
			D2 (?)	High concentrations: Induce the release of a-granules [61]

EPI: Epinephrine; NE: Norepinephrine; DA: Dopamine; D1/D2: Dopamine Receptors; ADP: Adenosine diphosphate; PF: Platelet Factor 4. ?: still under debate.

Despite NE induces platelet activation by binding, like EPI, α2-adrenergic receptors, its action is two or three times less effective than EPI [52,60] (Table 1).

Finally, dopamine potentiates platelet microaggregate formation and adhesion to collagen under low shear flow induced by ADP via D2-like receptor [58] (Table 1), however dopamine infusion in hypertensive and normotensive men do not influence platelet count, platelet size and plasma concentration of β-thromboglobulin [69].

Overall these data suggest that the inappropriate activation of the sympathoadrenal axis occurring under depression may increase the sensitivity of circulating platelets to agonists with severe consequences on CVD outcome.

## 3. Adipokines

Neuroendocrine regulators of energy metabolism are crucial in determining cardiovascular risk [70], and are associated with depression disorders [71]. In this contest, adipose tissue plays an endocrine role by synthesizing and secreting bioactive compounds named adipokines, whose secretion is essential to energy and metabolic homeostasis [72]. The most studied adipokines are leptin and adiponectin, whose alteration is reflected on both neuronal [73] and cardiovascular alterations [74,75]. Of note, among classical adipokines, also non-conventional metabolic regulators, like neurotrophins, could play a pivotal role in influencing both these pathology [76].

### 3.1. Leptin

Leptin is a hormone mainly secreted by adipocytes, it is involved in the control of food intake [77] and its increased levels are associated to obesity [78]. The peripheral actions of leptin include stimulation of inflammatory reaction, oxidative stress, atherosclerosis and thrombosis, thus promoting endothelial dysfunction, arterial stiffness, development and vulnerability of atherosclerotic plaques [79]. Moreover, it has been reported that CHD patients have higher leptin levels compared to controls [80,81]. Its serum concentrations are increased after myocardial infarction (MI) [82], and its high levels are associated with an increased risk of cardiac death, ACS, non-fatal MI, stroke and hospitalization for congestive heart failure [83,84].

#### 3.1.1. Leptin in Depression

Modifications of leptin metabolism and its gene expression, as well as its receptor, have been reported among patients with mental health disorders, including depression [85], independently of drug treatment [86]. However, the relationship between circulating leptin levels and depression is under debate. Some authors stated that depression is associated with low circulating and brain leptin levels [87,88,89,90], suggesting a correlation between leptin levels and the depressive mood. This data are supported by the observation that administration of leptin exerts an antidepressant-like effect [91], through dopaminergic neurotransmission regulation in mesolimbic areas [92]. In particular, leptin reduces symptoms of depression and has an anxiolytic effect affecting the HPA [93,94], and stimulating brain-derived neurotrophic factor (BDNF) production and function [95,96,97]. In addition, the deletion of leptin receptor (LepRb) and its downregulation are associated with depression-like behavioral impairments, indicating that leptin-lepRb signaling is involved in the molecular mechanism of leptin antidepressant action [98,99].

On the other hand, some studies did not find any difference in the leptin levels between depressed patients and control group [100,101,102,103], or measured higher levels of leptin in depressed patients [104,105,106,107,108,109]. In addition, a positive association between circulating leptin levels and depressive symptoms [110], mild/moderate but not severe depression [104,111], and with self-reported depressive symptoms, especially in women [109], was recently identified. A marked sexual difference in leptin levels has been consistently reported, usually both healthy and depressed women have higher leptin levels than men [88,104,112]. Several reasons of these sex-discrepancies have been hypothesized, including: **(a)** the greater amount of subcutaneous and intra-abdominal adipose tissue in women **(b)** the difference in male and female eating behavior or upregulated leptin mRNA in proportionally larger adipocytes of females and **(c)** the testosterone levels, that inversely correlates with leptin levels [113].

In general, confounders such as time of blood sampling, age, Body Mass Index (BMI), gender-associated metabolic disturbances, medication history and clinical type and features of depressive disorders, might impact peripheral leptin levels, and thereby justify inconsistent results obtained [101,104,107,114,115].

The presence of atypical major depressive disorders (MDD) may be an additional explanation of this contradictory results. High concentrations of leptin are specifically associated with atypical MDD and with symptoms that represent the core features of the atypical subtype, whereas no association was found for the typical subtype or when considering the general diagnosis of MDD [106]. This finding is consistent with the hypothesis of a leptin resistance process which blunts leptin central action, despite increasing peripheral concentrations, and leads to hyper-leptinemia in obese subjects [106]. Data from a large international consortium identified that 15% of patients with atypical depression carried a higher number of genetic risk variants for increased BMI, leptin and C-reactive protein (CRP), meaning that atypical depression and obesity-related traits may be the two faces of the same syndrome [108].

Taken together, all these data indicate the necessity of further investigation about circulating leptin levels in depressive disorders to understand its real impact on cardiovascular risk and thrombosis.

#### 3.1.2. Leptin and Platelet Function

Among other receptors, platelets express on their surface also receptors for peptide hormones, including the long form of leptin receptor (LEPRL) [116], suggesting that alteration of leptin levels occurring in depressive disorders may alter platelet response contributing to cardiovascular complications. Indeed, leptin promotes arterial thrombosis, potentiates platelet aggregation in mouse [117,118], increases platelets adhesion and potentiates ADP- and thrombin-induced aggregation in human [119,120], even if such effect was not observed in all subjects [116,117,118,121,122,123].

Furtherly, it has been shown that leptin induces platelet activation through almost two different signaling cascade mechanisms. The first includes the activation of Janus kinase 2 (JAK2), phosphatidylinositol 3-kinase (PI3K), protein kinase B (PKB), insulin receptor substrate-1 (IRS-1), and phosphodiesterase 3A (PDE3A), with a consequent increase of PDE3A and a decrease of cAMP [119,120]. The second one leads to GPIIb/IIIa activation, increase of Ca^2+^ and TX production through the activation of phospholipase C γ2 (PLCγ2), protein kinase C (PKC), and phospholipase A_2_ (PLA_2_) pathway [124].

### 3.2. Adiponectin

Adiponectin is an anti-inflammatory adipokine and contributes to increase insulin sensitivity protecting, therefore, against diabetes, atherosclerosis and thrombosis. Accordingly, high concentrations of adiponectin have been associated with a reduction in the risk of CVD [125,126,127] and an increase in endothelial nitric oxide production [128]. Conversely, patients with CVD, with increased carotid intima–media thickness and with obesity, exhibit low plasma adiponectin levels [129].

#### 3.2.1. Adiponectin in Depression

The relationship and the modulation of adiponectin in depressive disorders have been extensively studied. Specifically, several studies and a recent meta-analysis showed that MDD patients have low adiponectin levels [105,130,131,132,133,134], and that successfully antidepressant treatment increases its levels [135]. Interestingly, an inverse correlation between adiponectin levels and Hamilton Depression Rating Scale (HAM-D), indicating the depression symptoms severity at admission [130,132,136], or cumulative duration of depression [137] was identified. However, other studies were unable to confirm this observation [103,138,139,140]. Again, several confounding factors have to be taken into consideration. It is well known that (**a**) ethnic difference [140,141] between Asians and Europeans due to different body composition and metabolic profile [142], (**b**) matrix in which adiponectin is measured (plasma or serum) [134], (**c**) sex-dependent adiponectin levels [132,143], (**d**) body weight [137], (**e**) presence of metabolic syndrome [144], (**f**) onset of depressive event [107], and (**g**) the subtypes of depressive disorders [133,145,146,147] influence adiponectin levels.

However, when the modification of adiponectin levels are presents, they may contribute to platelet activation and then to thrombosis.

#### 3.2.2. Adiponectin and Platelet Function

Concerning the impact of adiponectin on platelet function, both mice and human platelets express adiponectin receptors AdipoR1 and AdipoR2 [124]. Adiponectin alone does not affect platelet adhesion/aggregation in human [120], nevertheless its deletion in a mouse model increases agonist-induced platelet aggregation and enhances thrombus formation after photochemically-induced arterial injury [148]. The antithrombotic effect of adiponectin may be related to its ability to influence leukocytes behavior, to reduce polymorphonuclear (PMN) leukocyte- and monocyte-platelet aggregates [149], and to inhibit macrophage-related Tissue Factor (TF) expression and activity with the consequent impairment in the coagulation cascade [150].

### 3.3. Neurothrophins

The neurotrophin (NT) family consists of Nerve Growth Factor (NGF), BDNF, NT-3 and NT-4 (also named NT-4/5). NTs not only are stimulators of nerve growth, survival and differentiation [151] but they also exert effects on immune cells [152,153,154], blood vessels/angiogenesis [155,156], wound healing and tissue repair [156,157], and most importantly on glucose, lipid and energy dynamics [158,159,160,161]. They are considered key regulators of metabolism [162], and for their pleiotropic functions, NTs have, as consequence, a huge importance in both depression [76], and CVD [76].

BDNF influences endothelial function [163], monocyte activation [154], and thrombus dimension and stability [164]. It takes part to cardiovascular development [165] but also to the onset of cardiovascular alterations and disease [166], including hypertension [167,168], atherosclerosis [169,170] and thrombosis. Reduced BDNF plasma levels has been found in metabolic syndrome [170], ACS [171,172], and type 2 diabetes [173], suggesting that alterations in its circulating levels may be associated to pathological conditions.

NGF and NT-3, similarly to BDNF, are involved in the cardiac development [174] and regeneration [175], angiogenetic process [165,176], hypertension onset [76,177], and atherosclerotic lesions formation [170,172,178].

The role of NT-4 and related pathways in CVD are not well characterized; however, binding the same receptor of BDNF (Tropomyosin receptor kinase B-TrkB), it might have similar functions of BDNF in controlling blood pressure [168].

Interestingly, several NT polymorphisms, including BDNF rs6265 (Val66Met) [179,180,181,182,183], NGF rs11102930 [184] and rs78701042 [185], have been associated to increased risk of ACS and adverse outcome.

#### 3.3.1. Neurotrophins in Depression

By their nature NTs play a key role in preventing depressive disorders [186], and data obtained in rodent models of depression indicate that administration of BDNF and NGF have significant antidepressant effects [187,188]. NTs do not control directly mood but, they are fundamental in the activity-dependent modulation of networks and changes in plasticity [11]. Low NT levels have been associated with several affective disorders including bipolar depression (BD) [189,190], depression [12,191], mania [192], and obsessive compulsive disorders [193]. In particular, reductions in serum and plasma BDNF have been found in patients affected by depression [194,195,196], and in those who committed suicide [197,198]. Despite a large cohort study [199] and meta-analyses [12,200,201] confirmed these results, there are a studies that have not found any relationship between BDNF and depression [202], or that provided evidence of positive correlation between BDNF and higher scores of scales for assessing depression in specific subgroups of patients [203]. Interestingly, genetic and epigenetic modifications in the BDNF gene have been associated to depressive disorders [204].

Similarly, alteration in circulating NGF levels have been detected in patients with depression. Clinical studies showed its reduced levels in patients with MDD compared to healthy control, and that NGF is negatively associated with depressive symptoms [205,206]. Nevertheless, in elderly patients this association seems to be attenuated [207].

As regard to NT-3 and 4/5 results are still controversial. Some studies found increased circulating levels of NT-3 [208,209], and NT-4/5 [209,210] in BD patients, while other studies demonstrated reduced [189,211] or unchanged levels [212,213].

#### 3.3.2. Neurotrophins and Platelets Function

The impact of NTs on platelet function has not been well-investigated yet. To our knowledge, it is only known that NGF can bind platelet surface and induce aggregation [214], while no information is available on the effect of BDNF on platelet function, even though BDNF can bind a specific site on platelets surface with subsequent internalization [215]. Platelets contain both NGF and BDNF, that are spontaneously released upon platelet activation [215,216,217,218,219]. From data present in literature is conceivable that NGF and BDNF are not stored in the same granules and that their release is mediated by different mechanisms, even though these mechanisms are still poorly characterized [219].

In particular, NGF is released within 10 min under calcium-free conditions, while after 60 min in presence of calcium his release is significantly reduced [219]. This event is faster when platelets are co-incubated with ibuprofen, while indomethacin do not affect NGF release [219].

On the other hand, only a low amount BDNF, stored in the α-granules [215], is released under calcium-free conditions, while calcium markedly increases its release. Ibuprofen and clopidogrel but not indomethacin and aspirin decrease its release [219,220]. Interestingly, when platelets are stimulated with thrombin, the mechanism of BDNF release is consequent to the activation of Protease-Activated receptor-1 (PAR-1). PAR-1 peptide induces a biphasic BDNF release, where only the first phase is calcium mobilization-dependent, as provided by its inhibition mediated by Prostaglandin-1 (PGE-1) pretreatment [217]. In this contest, authors demonstrated that BDNF is released by the fusion of α-granules with the Open Canalicular System (OCS), forming the swollen OCS [217].

**Figure 2:**
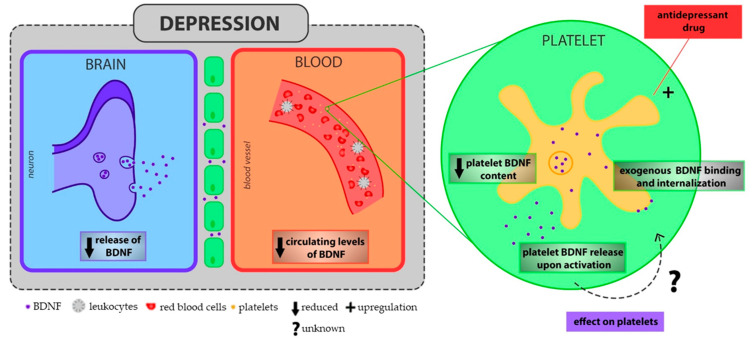
The controversial role of BDNF on platelet function. Depressed patients are characterized by the reduction of BDNF levels in the brain, that is well reflected by its decreased plasma and serum levels [221,222,223]. Remarkably, platelet BDNF content is reduced in depressed patients [224], but antidepressant treatment is able to restore their levels [224], indicating that a pre-activated state may promote platelet emptying of their BDNF reservoir. Platelets, when activated, can release BDNF [154,215,217,218], and extracellular BDNF can bind a specific site on platelets surface with subsequent internalization [215]. However, there are no information about its possible effect on platelets. BDNF: Brain-derived Neurotrophic Factor.

Interestingly, it has been shown that the amount of serum BDNF well reflect the amount of BDNF found in platelets [221]. In agreement with the decreased levels of BDNF under depression conditions [222,223], the platelet BDNF content is significantly reduced in patients with depression compared to control subjects [224], and the antidepressant pharmacotherapy normalize its levels. Then, it is possible to speculate that the platelet pre-activation state of MDD patients leads to their BDNF reservoir depletion [224], influencing negatively endothelial function and thrombus growing and stability [154,164,166] (Figure 2).

The genetic knock-in mouse carrying BDNFVal66Met polymorphisms, that recapitulates the phenotypic hallmarks of human disease (e.g., depression and CVD) [179] has been a helpful model to investigate the relationship between this polymorphism and platelet function. Specifically, the presence of this polymorphism predisposes to platelet hyper-activated phenotype, enhancing P-selectin expression, GPIIb/IIIa receptor activity, ability to bind leukocytes and fibrinogen, and aggregation [179,225].

## 4. Lipid Molecules and Lipoproteins

Lipids are essential structural components of cell membranes and play a crucial role in different metabolic pathways and cellular functions [226]. Lipids alteration is associated with cardiovascular risk [70]. A rise in cholesterol concentration increases the risk for death by CVD [227], and low-density lipoprotein (LDL), contributing to the development of atherosclerotic plaques, are included among the traditional risk factors for thrombosis. LDL may be oxidized, entrapped in macrophages inducing their differentiation in foam cells, and they may also bind to proteoglycans in the arterial intima. In addition to the critical role of LDL, a reduction of high-density lipoproteins (HDL) may cooperate to promote atherosclerosis. HDL inhibits oxidation of LDL, removes cholesterol from foam cells and reduces inflammation [228,229].

### 4.1. Lipid, Low Density Lipoprotein and Lipids Peroxidation in Depression

A large body of literature has provided knowledge on the relation between lipid status and psychotic disorders [230,231,232], whereas very few data is available on depression.

Nevertheless, a relation between depression and lipid disturbances has been recently demonstrated [233,234,235,236,237,238,239,240].

Actually, high total cholesterol (TC) and LDL are significantly associated with depressive symptoms and severity, with depression prospective course [236], and with metabolic syndrome in MDD [237]. In support of the relation between the severity of depression and cholesterol alteration, Enko et al. showed that, although depressed patients display only a slight increase in TC and LDL, there is a positive correlation between BDI-II depression score and triglycerides (TG), TC and LDL, and a negative, even though not significant, correlation with HDL [238]. In particular, an increase in LDL/HDL ratio has been observed in MDD patients [235,239,240]. By contrast, other studies showed that lower concentrations of TC and LDL were associated with MDD [241,242], and with incidence of depression [243,244,245,246], and that during first episodes of MDD higher TG levels and low HDL but similar LDL levels were found [247]. In addition, emerging data from a meta-analysis showed that depression was inversely associated with TC levels, and directly related to HDL levels, especially in women [235], whereas a U-shaped relationship with LDL was found [234]. The cross sectional nature of most studies, the different categorization of age, sampling and dissimilar tools of evaluation of depression applied in the studies, as well as the considerations of confounder factors (e.g., BMI, drug treatments) might explain the controversial findings about depression and circulating lipids and lipoproteins. Moreover, several studies analyzed the association of metabolic alterations with MDD, without carrying information about the inclusion of the BD individuals or without specifying which episode they were experiencing [237], all factors that could affect the conclusions.

Additionally to lipid molecules, the potential relationship with lipid peroxidation products as well as with oxidized LDL (oxLDL) and depression have to be considered [248].

The lipid peroxidation marker malondialdehyde (MDA) is increased in MDD patients compared to healthy control [249,250,251,252], and correlates with depression severity [253,254]. Similarly, the metabolites of F2 isoprostanes, an additional marker of lipid peroxidation, are greater in urine, plasma and serum of patients with depressive disturbances or MDD. This difference is particularly marked in elderly men [255], and is sex- and age-independent [256]. Only one study did not find significant difference between depressed and control subjects; however, this conclusion may be a result of the small sample size used [257].

Depressed patients have also higher levels of serum oxLDL antibodies than normal control [258], and a positive correlation emerged between serum oxLDL/LDL ratio with both Centre Epidemiological Studies Depression Scale (CES-D) score and perceived stress in a Japanese population [259]. Interestingly, depressed patients with great serum oxLDL antibodies are at high risk for atherosclerosis or have atherosclerotic lesions [9,258], suggesting a relationship among depression, oxLDL and CVD.

Overall, the positive association found between LDL or oxLDL and BMI [260,261], and between BMI and depressive symptoms [71], suggests that depression, directly or indirectly, leading to weight gain and to metabolic syndrome, could predispose to lipid alteration and to CVD.

### 4.2. Low Density Lipoprotein, Lipids Peroxidation and Platelet Function

Disorders in lipid status, above described, in patients with depression and CVD may contribute to platelets activation leading to acute thrombotic events.

LDL increases the sensitivity of blood platelets to agonist stimulation, making their response faster and more extensive [228]. Individuals with high plasma-LDL have hyper-reactive platelets and greater plasma levels of platelet activation markers, including BTG and soluble CD40 ligand (CD40L) [228]. In vitro, platelets exposed to LDL display hyper-aggregability, increased fibrinogen binding and surface-expression of P-selectin, and increased production of TX, and generation of Reactive Oxygen Species (ROS) [228,262].

Of note, oxLDL can influence platelet activation, apoptosis, and association with monocytes/macrophages [228]. In particular, by binding scavenger receptors on platelets (e.g., CD36) [228,263], oxLDL enhances NADPH oxidase-2 (NOX-2)-mediated generation of ROS and platelet hyper-reactivity [264], including platelet degranulation, GPaIIb3-integrin activation, apoptosis, thrombin generation, and shape change. Finally, LDL–oxLDL enhances platelet release of CXCL12 (Stromal Cell-derived Factor-1-SDF-1), that in turn prompts LDL–oxLDL uptake and synergistically augments the LDL–oxLDL-induced pro-oxidative and thrombogenic impact on platelet function [265].

## 5. Reactive Oxygen Species

ROS (i.e., superoxide anion (O•_2_-), hydrogen peroxide (H_2_O_2_), hydroxyl radical (•OH), hydroxyl ion (OH−) consist of radical and non-radical oxygen species formed by the partial reduction of oxygen [266]. ROS modulate several physiological processes, however when their excessive production is not counteracted by antioxidant capacity of human physiology there is imbalance in the redox system, resulting in tissue damage and in the development and progression of several diseases. The implication of ROS in the pathogenesis of CVDs and thrombosis has been well described [267]. Abnormal ROS increase has been observed in atherosclerosis [268], and in coronary artery disease (CAD) patients and associated with future CVD events [269]. The most well-known sources of ROS in the cardiovascular system are NOX family of enzymes, uncoupled endothelial nitric oxide synthase (eNOS), mitochondria and xanthine oxidase (XO), whose function is critical in determining the onset and progression of CVD [270].

### 5.1. Reactive Oxygen Species in Depression

Clinical studies have reviewed the possible impact of oxidative stress in the pathophysiology of depression [271,272,273,274], focusing on ROS iper-production or on the activation of enzymes relevant in pro/antioxidant processes [e.g., NOX, XO, superoxide dismutase (SOD) and catalase (CAT)] [274], and experimental models have established that the enhanced ROS production favors depression-like phenotype [275].

Several peripheral markers of oxidative stress and mechanisms implicated in redox balance are altered in MDD, nevertheless, there is a significant heterogeneity across the studies [276], specifically as regard to ROS alteration [277], hence importance of careful phenotyping of the depressed and control subjects.

Decreased SOD activity [253,278] in red blood cells (RBC) of depressed patients have been measured in first [253], recurrent [251], and bipolar episodes [279] suggesting also a connection with different subtypes of depressive manifestations.

**Figure 3:**
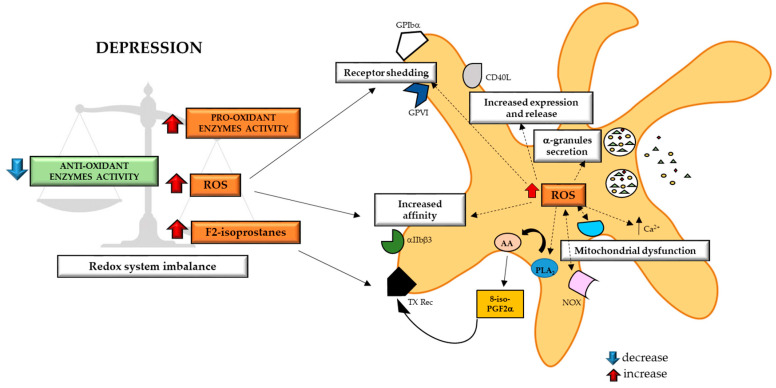
Increased oxidative stress that occurs during major depressive disorders (MDD), can activate platelets. Depressed patients are characterized by an imbalance in redox system with increased pro-oxidant enzyme activity not counterbalanced by anti-oxidant enzyme [251,252,253,276,278,279]. This imbalance promotes an excessive production of ROS and an increase in F2 isoprostanes circulating levels. Extracellular ROS is then able to activate platelets increasing GPIIb/IIIa receptor affinity, and inducing GPIα and GPVI receptors shedding with consequent activation of downstream pathways [282,283]. In the same time, the activation of platelets due to pro-oxidant environment favors intra-platelets production of ROS. Intra-platelets ROS support platelet activation promoting α-granule exocytosis, increasing the sensitivity of platelet receptors, acting as second messenger in thrombin- or collagen-activated platelets, inducing calcium mobilization, upregulating CD40L surface expression and release and generating isoprostanes, including 8-iso-PGF2α [activating AA metabolism] [290,291,292,293,294]. PGF2α can in turn activates TX receptor beyond supporting platelet activation [295,296,297]. Finally, the redox imbalance is furtherly fed by a vicious cycle of ROS production due to activation of NOX enzyme [285,286,287,288,289] and to alteration of platelet mitochondrial function [298,299]. ROS: Reactive Oxygen Species; GP: glycoprotein; CD40L: CD40 Ligand; Ca^2+^: Calcium; NOX: NADPH oxidase; PLA_2_: Phospholipase A_2_; AA: Arachidonic Acid; TX: Thromboxane.

The reduction of SOD activity associated with increased XO activity [278] and unchanged CAT activity [253,276] well explain the increased ROS generation detected in patients with depression [252,276]. However, other studies showed increased RBC SOD activity [249,250,252], that is potentially explained as a protective mechanism induced by organism to counterbalance oxidative stress that occurs during depressive disorders (Figure 3).

A recent meta-analysis indicated that depression is associated with enhanced oxidative damage, as provided by increased urinary and serum/plasma levels of 8-hidroxy-2′-deoxyguanosine (8-OHdG) and F2-isoprostanes [273] (Figure 3).

Noteworthy is that mitochondrial dysfunction is considered the preferential mechanism as source of ROS in MDD [280,281]; indeed, reduced mitochondrial function is implicated in depression onset and progression [281]. This finding is relevant since the brain is a highly active organ with high energy consumption, and then it is more susceptible to the deleterious effect of excessive ROS production related to mitochondrial dysfunctions.

Overall, the imbalance of redox system, with an increased production of ROS, that characterizes depression disorders may cover a key role in promoting platelets activation.

### 5.2. Reactive Oxygen Species and Platelet Function

Extracellular ROS promote the activation of GPIIb/IIIa interacting with thiol groups in the extracellular domain, and the shedding of GPVI and GPIbα through a mechanism mediated by A Disintegrin and Metalloproteases (ADAM) [282,283]. These events have been recently associated with increased coagulation factor binding and enhanced thrombin and fibrin generation, favoring a pro-coagulant phenotype of platelets [284]. Activated platelets, via NOX, cyclooxygenases, eNOS, XO, and mitochondrial respiration [285,286], are able to generate *per se* ROS, that in turn re-activate platelets [287,288], especially in older patients [289]. As consequence, intra-platelets ROS support platelet activation promoting α-granule exocytosis [290], increasing the sensitivity of platelets receptor like GPIIb/IIIa, GPIbα and GPVI [291,292]. When platelets are exposed to thrombin-or collagen ROS act as second messenger [293], inducing calcium mobilization [293], upregulating CD40L surface expression and release [294], and generating isoprostanes including 8-iso-prostaglandin F2α (PGF2α) that can promote platelet aggregation via TX receptor in the presence of low concentrations of other agonists [295,296,297] (Figure 3).

Interestingly, alteration in platelets mitochondrial bioenergetics has been detected in MDD patients compared to a matched control subjects [298,299], supporting the hypothesis of interplay among ROS, platelet activation and depression.

## 6. Inflammatory Factors

Inflammatory process has been extensively described in worsening CVD prognosis, and platelets are recognized as active mediators of this mechanisms [300]. The pro-inflammatory status has been also associated with MDD and is exhaustively described in literature. In particular, pro-inflammatory cytokines like interleukin-2 (IL-2), IL-6, soluble IL-6 receptor, tumor necrosis factor-α (TNF-α) and interferon-γ (IFN-γ) are increased while anti-inflammatory cytokines like IL-4 and IL-10 are decreased during depressive disorders [301]. More recent studies demonstrated that also chemokines contribute to neurobiological processes relevant to psychiatric disorders [302], suggesting another point of connection between depression and cardiovascular disease.

### 6.1. Chemokines

Chemokines are small (8–12 kDa) chemotactic cytokines, which have an important role in directing the migration of blood cells to target tissues. Chemokines are classified into 4 groups, with the CC- and CXC-types being the most common [303]. Alteration in circulating levels of chemokines like CXCL8 (IL-8), CCL2 (Monocyte Chemoattractant Protein-1 (MCP-1), CCL26 (Eosinophil Chemotactic Protein-3 (Eotaxin-3), CCL5 (Regulated on Activation of Normal T cell-expressed and secreted (RANTES) entities) CXCL10 (γ-Interferon-inducible Protein-*10* (IP-10) or of chemokines receptors (e.g., fractalkine receptor-CX_3_CR1) are associated with subclinical or proclaimed cardiovascular pathology (e.g., MI, CAD, atherosclerosis) or with cardiovascular death [304,305,306]. In addition, it has been showed that the fractalkine receptor CX_3_CR1 plays a key role in atherosclerosis [305], and that a polymorphism in its gene is associated with a reduced risk for CAD [306].

Overall, chemokines represent a promising therapeutic target in cardiovascular disease [303], and recently have been also related to depression disorders [307].

#### 6.1.1. Chemokines in Depression

Several studies highlighted the link between depressive symptoms and elevated circulating levels of chemokines, and the main results obtained are summarized in a recent review [307].

Higher blood levels of CCL11 (Eotaxin-1), RANTES, SDF-1 and CXC_3_L1 (fractalkine) were found in depressed patients compared to controls [308,309,310,311] (Table 2).

In particular, Ogłodek et al. showed that RANTES and CXCL12 levels were significantly increased in both women and men with depressive disorders and that a relation between circulating levels of these chemokines and severity of depressive symptoms exists [309]. By contrast, Leighton et al., in a meta-analysis including 7 studies, did not find significant difference in circulating levels of RANTES and CCL3 (Macrophage Inflammatory Protein-1α (MIP-1α) between MDD patients and control group [307] (Table 2). However, need to be mentioned that the conclusion of Leighton et al. may be incorrect due to the inclusion of studies that evaluated indiscriminately the RANTES levels in plasma, serum and whole blood of depressed patients (Table 2). Indeed, it has been reported that depressed patients have reduced serum levels of RANTES [308] and antidepressant treatment can restore its levels [311]. Nevertheless, reduced RANTES serum levels in depressed patients may be related to the activated platelet phenotype, that leads to the emptying of RANTES platelet reservoir [312].

Increased plasma levels of fractalkine in moderate-severe depressed patients compared to control subjects was found [313] and this result was confirmed by other studies, in which a correlation between fractalkine plasma levels and the severity of depressive symptoms was identified [314,315] (Table 2). However, considering the scarcity of information about circulating levels of fractalkine in association with depression, further studies are needed, also taking into account the possible confounding factors (e.g., age, BMI) and the heterogeneity of MDD.

Instead, CCL4 (MIP-1β) has been found lower in the serum of depressed patients compared to not depressed ones [307].

Finally, MCP-1 levels are usually higher in depressed patients compared to controls [302,310,311,316,317], with the exception of some works [131,318] (Table 2). The careful analyses of these works highlighted that patient groups are highly heterogeneous, including different subgroups of depressed patients, e.g., patients with bipolar disturbs [316], obsessive-convulsive disorders [319], exhaustion [320], and nocturnal disturbs [321], leading to find no difference, almost in all cases, in circulating levels of MCP-1. Indeed, it is showed that MDD patients with suicidal ideation have surprisingly reduced levels of this chemokine [308].

In conclusion, the studies investigating the relationship between chemokines and depression are still few and often controversial, more research is needed to define whether their circulating levels are really enhanced in depression.

#### 6.1.2. Chemokines and Platelet Function

The presence of chemokine receptors on the surface of platelets has been controversial for a long time, but now accumulated evidence show that CXCR4, CCR4, CX_3_CR1 and lower but still functional amounts of CCR1 and CCR3 are present on platelets [322,323,324,325].

As regard of chemokines whose levels result altered in MDD, only few were investigated in relation to platelets function. Nevertheless, some of them (e.g., MCP-1, Eotaxin-1, SDF-1 and fractalkine) are promising mediators of the link between depression and platelets activation.

It has been demonstrated that the activation of CCR1, 3 and 4 receptors by specific chemokines, including MCP-1, MIP-1α, Eotaxin, and RANTES, induces platelet aggregation and release of platelet granule contents [325] (Table 2).

In addition, fractalkine, through the activation of CX_3_CR1, promotes adhesion of platelets on fibrinogen and collagen [326], and induces not only monocyte recruitment but also platelet accumulation at sites of arterial injury [327]. It has been recently demonstrated that also GPIb can acts as a fractalkine receptor [328], thus suggesting another possible target in regulating platelets activation. Interestingly, a positive correlation between levels of fractalkine and platelet activation in patients with CVD was identified [326] (Table 2).

Finally, SDF-1, expressed on both megakaryocytes and platelets [322], modulates megakaryocytes maturation and megakaryocytopoiesis [329], induces platelets activation, enhancing platelet aggregation and intra-platelet Ca^2+^ flux, and it modulates the expression of CXCR4-CXCR7 receptors on platelets surface [330]. Of note, the expression of CXCR4-CXCR7 receptors on platelets have a prognostic value in CVD [330] (Table 2).

**Table 2:** Chemokines potentially relevant in the relationship between depression and platelet activation.

DEPRESSION			PLATELETS	
**Chemokines**	**District**	**Levels**	**Receptor**	**Effect**
MCP-1	Serum Plasma	↑ [302,310,311,316,317]	CCR1/CCR3	Platelet aggregation [325] Granules content release [325]
Eotaxin-1	Serum	↑ [308,310]	CCR1/CCR3	Platelet aggregation [325] Granules content release [325]
RANTES	Serum Plasma	↓ [307,308,311] ↑/= [307,309]	CCR1/CCR3	Platelet aggregation [325] Granules content release [325]
SDF-1	Plasma	↑ [309]	CXCR4	Megakaryocytes maturation and megakaryopoiesis [329] Platelets activation, aggregation and Ca^2+^ flux [330] Expression of platelets surface expression of CXCR4-CXCR7 receptors [330]
MIP-1α	Plasma	= [307,311]	CCR1/CCR3	Platelet aggregation [325] Granules content release [325]
MIP-1β	Serum	↓ [307]	CCR1/CCR3	NA
Fractalkine	Plasma	↑ [313,314,315]	CXC_3_CR1	Platelet adhesion [326] Platelet accumulation and monocytes recruitment at injury site [327] Platelet activation in CVD patients [326]

↑: increased, ↓: decreased; =: similar levels compared to control. MCP1: Monocyte Chemoattractant Protein-1; Eotaxin-1: Eosinophil Chemotactic Protein-1; RANTES: Regulated on Activation of Normal T cell-expressed and secreted; SDF-1: Stromal Cell-derived Factor-1; MIP-1α/β: Macrophage Inflammatory Protein-1α/β.

Interestingly, platelets store and release several chemokines [331] that influence both physiological and pathological conditions [332]. To our knowledge, the relationship among chemokines platelet-released, depression and CVD has not been investigated as yet. Nevertheless, we can hypothesize that chemokines released by hyper-reactive platelets contribute to progression of both depression and CVD, worsening the clinical outcome.

## 7. Conclusions

In spite of the provided evidence about highest prevalence of CVD in patients with depression, the molecular mechanisms at the basis of this relation are largely unknown. The hyper-activated response of platelets of depressed patients suggest them as possible and interesting mediators in this contest.

In this review we critically analyzed some of the molecules, whose alteration is relevant in promoting the onset of CVD and that are known or potentially associated with platelets activation and de-/activation, in depressive disorders. Even if many of the studies taken into consideration seem to suggest that circulating levels of these molecules are changed during depression, consequently favoring platelets activation, the results present in literature are often still controversial and need further investigation to give a univocal indication. In this perspective, it will be useful to study the pathways activated by these molecules in platelets of depressed patients to have an exhaustive insight about this subject matter and to understand molecular mechanisms underlying the link among depression, platelet activation and CVD.

## Abbreviations

5-HT	5-Hydroxytriptamine
5-HT2	5-HT receptor
8-isoPGF2α	8-iso-prostaglandin F2α
OHdG	8-hydroxy-2′-deoxyguanosine
ACS	Acute Coronary Syndrome
ADAM	A Disintegrin and Metalloproteases
ADP	Adenosine Diphosphate
AR	Adrenergic Receptor
BD	Bipolar Depression
BDNF	Brain-derived Neurotrophic Factor
BMI	Body Mass Index
BTG	β-thromboglobulin
CAD	Coronary Artery Disease
cAMP	3’-5’-Cyclic Adenosine Monophosphate
CAT	Catalase
CD40L	CD40 Ligand
CES-D	Centre Epidemiological Studies Depression Scale
CHD	Coronary Heart Disease
CRP	C-Reactive Protein
CVD	Cardiovascular Disease
CXC_3_L1	Fractalkine
CXC_3_R1	Fractalkine Receptor
DA	Dopamine
eNOS	Endothelial Nitric Oxide Synthase
EPI	Epinephrine
Eotaxin	Eosinophil Chemotactic Protein
G protein	Guanine nucleotide-binding proteins
GP	Glycoprotein
HAM-D	Hamilton Depression Rating Scale
HDL	High-Density Lipoprotein
HF	Heart Failure
HPA	Hypothalamic-Pituitary-Adrenal
IFN-γ	Interferon-γ
IL	Interleukin
IP-10	γ-Interferon-inducible Protein-10
IRS-1	Insulin Receptor Substrate-1
JAK2	Janus Kinase 2
LepRb	Leptin Receptor
LEPRL	Long form of Leptin Receptor
LDL	Low-Density Lipoprotein
MCP-1	Monocyte Chemoattractant Protein-1
MDA	Malondialdheyde
MDD	Major Depressive Disorders
MI	Myocardial Infarction
MIP-1α/β	Macrophage Inflammatory Protein-1α/β
NE	Norepinephrine
NGF	Nerve Growth Factor
NOX	NADPH oxidase
NT	Neurotrophin
OCS	Open Canalicular System
oxLDL	Oxidized LDL
PAR-1	Protease-Activated Receptor-1
PDE3A	Phosphodiesterase 3A
PF4	Platelet Factor 4
PGE-1	Prostagladin E-1
PLA/C	Phospholipase A/C
PI3K	Phosphatidylinositol 3-Kinase
PK	Protein Kinase
PMN	Polymorphonuclear
RANTES	Regulated on Activation of Normal T cell-expressed and secreted
RBC	Red Blood Cells
ROS	Reactive Oxygen Species
SDF-1	Stromal Cell-derived Factor-1
SOD	Superoxide dismutase
TC	Total Cholesterol
TF	Tissue Factor
TG	Triglycerides
TNF-α	Tumor Necrosis Factor-α
TrkB	Tropomyosin receptor kinase B
TX	Thromboxane
vWF	Von Willebrand Factor
XO	Xantine oxidase
